# Equilibria in Aqueous Cobalt(II)—Reduced Schiff Base *N*-(2-hydroxybenzyl)alanine System: Chemical Characterization, Kinetic Analysis, Antimicrobial and Cytotoxic Properties

**DOI:** 10.3390/molecules25153462

**Published:** 2020-07-30

**Authors:** Magdalena Woźniczka, Manas Sutradhar, Armando J. L. Pombeiro, Mirosława Świątek, Marek Pająk, Joanna Gądek-Sobczyńska, Magdalena Chmiela, Weronika Gonciarz, Beata Pasternak, Aleksander Kufelnicki

**Affiliations:** 1 Department of Physical and Biocoordination Chemistry, Faculty of Pharmacy, Medical University of Lodz, Muszyńskiego 1, 90-151 Lodz, Poland; miroslawa.swiatek@umed.lodz.pl (M.Ś.); marek.pajak@umed.lodz.pl (M.P.); joanna.gadek-sobczynska@umed.lodz.pl (J.G.-S.); aleksander.kufelnicki@umed.lodz.pl (A.K.); 2 Centro de Química Estrutural, Instituto Superior Técnico, Universidade de Lisboa, Av. Rovisco Pais, 1049-001 Lisboa, Portugal; manas@tecnico.ulisboa.pt (M.S.); pombeiro@ist.utl.pt (A.J.L.P.); 3 Department of Immunology and Infectious Biology, Institute of Microbiology, Biotechnology and Immunology, Faculty of Biology and Environmental Protection, University of Lodz, Banacha 12/16, 90-237 Lodz, Poland; magdalena.chmiela@biol.uni.lodz.pl (M.C.); weronika.gonciarz@biol.uni.lodz.pl (W.G.); 4 Department of Organic Chemistry, Faculty of Chemistry, University of Lodz, Tamka 12, 91-403 Lodz, Poland; beata.pasternak@chemia.uni.lodz.pl

**Keywords:** cobalt(II) complexes, coordination modes, reduced Schiff base, stability constant, biological activity, UV-Vis spectroscopy, ESI-MS

## Abstract

The present study describes the coordination properties of a reduced Schiff base, *N*-(2-hydroxybenzyl)alanine, towards cobalt(II) using potentiometric as well as spectroscopic (UV-Vis and ESI-MS) methods. The results indicate the formation of six mononuclear complexes showing high stability in aqueous solution. Coordination occurs in the {O^−^_phenolic_,N,O^−^_carboxyl_} and {N,O^−^_carboxyl_} chelation modes, depending on the degree of ligand deprotonation. Examination of the complexation equilibria at pH *ca* 7, which is important from a biological point of view, allowed to identify two species: [CoL] and [CoL_2_H]^−^. The kinetic analysis showed a structural change of those cobalt(II) complexes from octahedral to tetrahedral in accordance with a first-order time relationship. The antimicrobial properties of *N*-(2-hydroxybenzyl)alanine, cobalt(II) nitrate and of the Co(II) – ligand complexes were determined against Gram-positive bacteria (*Enterococcus faecalis*, *Staphylococcus aureus*, *Staphylococcus epidermidis*), Gram-negative bacteria (*Pseudomonas aeruginosa*, *Escherichia coli*, *Helicobacter pylori*) and a fungal strain (*Candida*). The results indicate that the complexes are more active for more strains than the ligand alone. Nevertheless, the complexes induce a higher decrease in the metabolic activity of cells but without damage to nuclei. Tetrahedral structures show stronger anti-cellular toxicity than octahedral complexes, which is most likely due to the higher accessibility of the cobalt(II) center.

## 1. Introduction

Schiff bases, or their reduced forms, can stabilize various transition metal complexes due to their strong chelating ability towards metal ions [1,2,3]. Ligands derived from amino acid Schiff bases are mainly tridentate, binding a metal ion via phenolic, imino and carboxyl groups in a {O,N,O} chelating mode. The reduced form of the Schiff base, derived from an amino acid and a carbonyl compound (e.g., salicylaldehyde), can form transition metal complexes with important applications [4,5,6]. Amino acids play important roles in various biochemical processes as endogenous ligands; in addition, their complexes with biologically-active metal ions have also proved to be useful antibacterial agents and metalloprotein models [7,8,9].

The presence of an imino group in amino acid Schiff bases can reduce stability. The reduction of the C=N bond to a single amine bond increases the conformational flexibility of the backbone, making the reduced Schiff bases more stable molecules, which can be used as models in biological reactions. They are also popular ligands for antiradical and antioxidant complexes [1,4]. In addition, Schiff base complexes exhibit anti-inflammatory [10], antibacterial [11,12], antifungal [13], anticarcinogenic [14,15,16,17,18], catecholase [19,20] activities and DNA-binding ability [21,22].

Microorganisms are demonstrating a growing accumulation of drug resistance, which has been attributed to biochemical and morphological modifications, especially in the case of bacteria, as well as failures in the development or discovery of new antibiotics and excessive use in the treatment of infectious diseases [23]. *Candida* infections (candidiasis) are the most prevalent opportunistic fungal infection in humans and, as such, constitute a major public health problem. In recent decades, candidiasis has been associated with *Candida* species other than *Candida albicans.* Moreover, biofilms have been considered the most prevalent growth form of *Candida* cells and a strong causative agent of the intensification of antifungal resistance. In turn, *C. albicans* is able to grow together with *Staphylococcus aureus*, *S. epidermidis* and *Enterococcus* spp. in the course of blood-borne infections, *Pseudomonas aeruginosa* in cystic fibrosis and with a range of other bacteria or fungi in oral and skin/wound infections [24,25,26].

The above problems have driven the search for new, alternative antimicrobial substances. In many cases, the formation of complexes with transition metals enhances the biological activity of ligands [14,15,16,27]; bio-essential metals are commonly used due to their growing role as therapeutic agents [28]. For example, in addition to being part of enzymes and vitamin B_12_, cobalt also demonstrates antibacterial, antifungal and antiviral activities [29,30]. Therefore, many studies focus on the synthesis of new metal complexes with reduced Schiff bases and the exploration of their structures due to promising biological functions [4,10].

**Figure 1:**
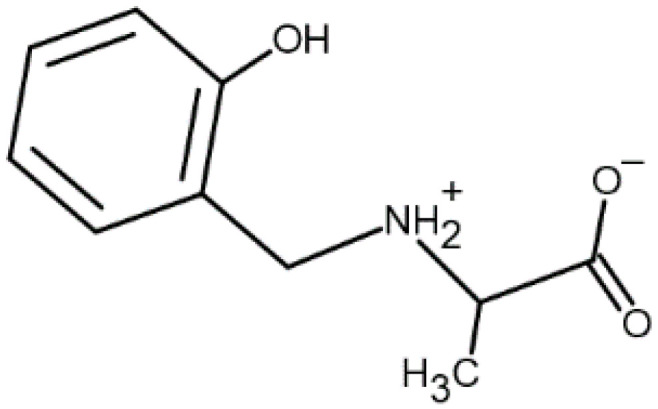
Structure of the ligand *N*-(2-hydroxybenzyl)alanine, AlaSal (zwitter-ionic form [LH_2_]).

The present study examines the complexation equilibria in the aqueous system of cobalt(II) with the reduced Schiff base *N*-(2-hydroxybenzyl)alanine, AlaSal (Figure 1), obtained as a result of a reaction between salicylaldehyde and L-alanine, followed by reduction. The formation of specific complex structures is strongly influenced by the nature of the ligands, the pH of the solution, the nature of solvents used, and the concentration of dioxygen in solution [20]. Therefore, in the initial stage of biological research into such compounds, it is necessary to determine the effect of pH on the types of complexes formed, and then their stability in aqueous solution. The evaluation of the antimicrobial properties of AlaSal, as well as of its cobalt(II) complexes present in the system at a pH of about 7 towards several reference bacterial and fungal pathogens was the next step of research. The cytotoxicity of the studied complexes was also examined in order to determine their safety for use for eukaryotic cells.

## 2. Results and Discussions

### 2.1. Protonation and Complex Formation Equilibria

The protonation constants of AlaSal, presented in Table 1, were determined in aqueous solution and show similar values as reported earlier [31]. The first dissociation constant, equal to 2.29, is assigned to the carboxyl group: the fully protonated form [LH_3_]^+^ becomes the zwitter-ionic [LH_2_]. In turn, the amine group (p*K*_a2_ = 8.62) deprotonates prior to the phenolic group (p*K*_a3_ = 10.72), i.e., the zwitter-ionic [LH_2_] becomes the [LH]^−^ form, and then the [L]^2−^ form after further deprotonation; this was also confirmed by some previous studies of hydroxybenzyl derivatives [32,33,34,35]. The individual ionic forms of the ligand are presented in the pH-dependent species distribution diagram (Figure S1).

**Figure 2:**
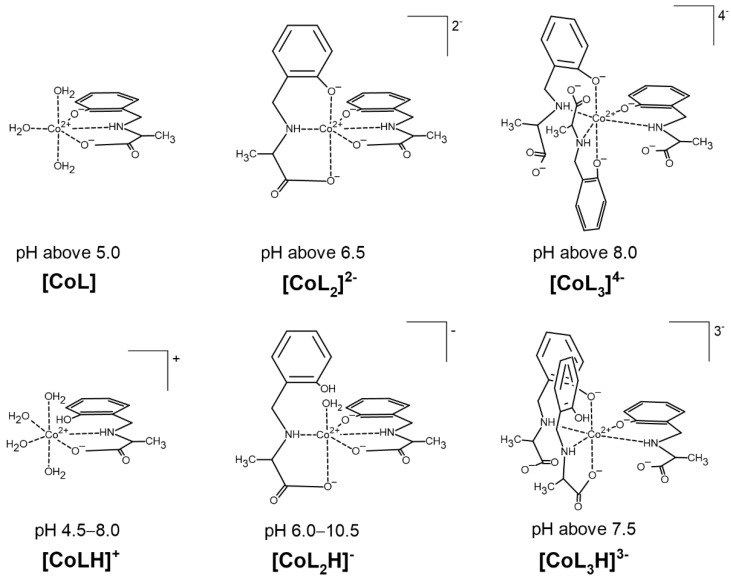
Suggested coordination modes of the complexes in the Co(II)–AlaSal system in dependence on the pH.

**Figure 3:**
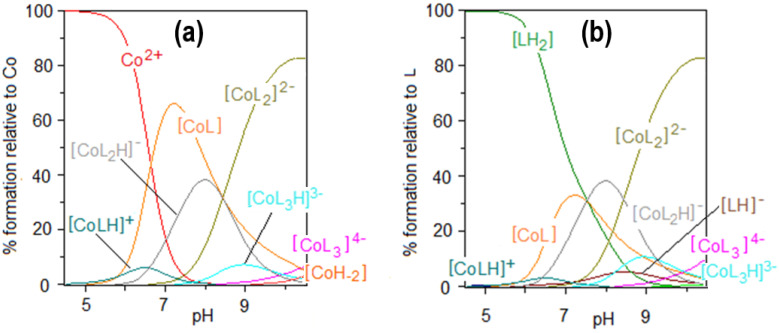
Species distribution curves for the complexes formed in the Co(II)–AlaSal system at ligand-to-metal molar ratio 2:1 as a function of pH relative to (**a**) Co(II), (**b**) ligand; C_AlaSal_ = 2.0 × 10^−2^ M.

The results of the potentiometric titrations in the Co(II)–AlaSal system confirm the formation of six complex species (Figure 2). The overall stability constants of these complexes are presented in Table 1. Complexation in Co(II)–AlaSal system has been previously described in the literature for divalent transition metals, such as Co, Ni, Cu and Zn [31]. Nevertheless, the current study in the presence of Co(II) indicates a higher number of complexes in the aqueous system. Based on the formation constants, a representative species distribution graph is presented for AlaSal-to-Co(II) at a 2:1 molar ratio (Figure 3). At a pH value of about 10.5, the precipitation became visible due to formation of the Co(II) aqua–hydroxo complex [CoH_−__2_].

The [CoL] complex, containing a completely deprotonated form of the ligand, is one of the most abundant species in the system. The tridentate form of AlaSal, showing a flexible conformation [36,37], most likely binds with the central ion in the equatorial sites via amine, phenolic and carboxyl groups and forms two chelate rings, five- and six-membered. This coordination mode is indicated by a higher value of the stability constant for the [CoL] (Table 1) than for the analogous complex with histidine (6.74), in which chelation of one donor atom occurs in the axial position [31,38]. Additionally, solid-state complexes obtained for AlaSal systems with transition metal ions showed the equatorial coordination of ligand [39,40,41]. In turn, as reported for analogous X-ray crystal forms [10,42], the equatorial plane for the bi-ligand complex is most likely occupied by one AlaSal molecule, indicating {O^−^_phenolic_, N,O^−^_carboxyl_} chelation mode and the nitrogen atom of another molecule. The other two oxygen atoms are probably weakly bound in axial positions [31]. Different planes of ligand molecules were previously confirmed by the lack of significant intramolecular ligand–ligand interaction between the non-coordinated side groups of Schiff base derivatives and amino acids [43]. In turn, an excess of donor groups relative to the coordination sites is observed in [CoL_3_]^4−^, which probably forces a different complexation mode in this structure than in earlier complexes. The equatorial positions are most likely occupied by three amine nitrogens and one phenolic oxygen. A similar coordination was also found in a previously described complex containing a reduced Schiff base [36]. The coordination sphere at the axial sites is completed by the phenolic groups of two other AlaSal molecules.

Potentiometric studies suggest that besides the fully deprotonated complexes: [CoL], [CoL_2_]^2−^ and [CoL_3_]^4−^, AlaSal forms three species with cobalt(II): [CoLH]^+^, [CoL_2_H]^−^ and [CoL_3_H]^3−^, where the ligand molecule has a protonated phenolic group (Figure 2). The formation of [CoLH]^+^ starts around pH 4.5 (Figure 3a). The dissociation mechanism of AlaSal (Table 1) promotes chelation of cobalt(II) in classical amino acid mode by the amine nitrogen and the carboxyl oxygen, most likely in the equatorial plane. [CoLH]^+^ demonstrated a lower related stability constant value than the completely deprotonated [CoL], indicating the formation of one five-membered chelate ring as a result of bidentate {N,O^−^_carboxyl_} coordination (Table 1). Moreover, this type of coordination is supported by X-ray data for the crystal forms of hydroxybenzyl derivative complexes in which the phenolic −OH group participates in an intramolecular NH^…^O hydrogen bond [44,45]. In addition, the same {N_amine_,O^−^_carboxyl_} chelation mode is suggested for protonated histidine complex, showing a value of related stability constant (2.32) of the same order as [CoLH]^+^ [38,46]. The deprotonation step of the [CoLH]^+^ complex occurs according to the Equation (1):

[CoLH]^+^ = [CoL] + H^+^ (1)

hence

$$\log_{10}K_{\mathrm{CoLH}}^{\mathrm{CoL}}=\log_{10}\beta_{\mathrm{CoLH}}-\log_{10}\beta_{\mathrm{CoL}}=13.64-7.98=5.66$$

reaches a lower value than the corresponding free ligand deprotonation constant for the phenolic proton (p*K*_a3_ = 10.73). Therefore, the metal-promoted deprotonation allows the formation of [CoL] at pH above 5 (Figure 3a). The deprotonations of other protonated complexes [CoL_2_H]^−^ and [CoL_3_H]^3−^ are represented by the Equations (2) and (3):

[CoL_2_H]^−^ = [CoL_2_]^2−^ + H^+^, (2)

where

$$\log_{10}K_{{\mathrm{CoL}}_{2}H}^{{\mathrm{CoL}}_{2}}=\log_{10}\beta_{{\mathrm{CoL}}_{2}H}-\log_{10}\beta_{{\mathrm{CoL}}_{2}}=21.78-13.35=8.43$$

and

[CoL_3_H]^3−^ = [CoL_3_]^4−^ + H^+^ (3)

where

$$\log_{10}K_{{\mathrm{CoL}}_{3}H}^{{\mathrm{CoL}}_{3}}=\log_{10}\beta_{{\mathrm{CoL}}_{3}H}-\log_{10}\beta_{{\mathrm{CoL}}_{3}}=26.35-16.39=9.96$$

Hence, $pK_{{\mathrm{CoL}}_{2}}^{{\mathrm{CoL}}_{2}H}$(8.43) and $pK_{{\mathrm{CoL}}_{3}}^{{\mathrm{CoL}}_{3}H}$(9.96) also reach lower values than p*K*_a3_ (Table 1) and by that mononuclear complexes [CoL_2_]^2−^ and [CoL_3_]^4−^ are formed above pH 6.5 and 8.0, respectively.

### 2.2. ESI-MS Results

The ESI-MS spectra for AlaSal and its system with Co(II) were recorded in order to determine the type of formed complexes and compare them with potentiometric results. The interpretation of the tandem mass spectrum (Figure S2) of the protonated ligand molecule [LH_3_]^+^ (*m*/*z* = 196.0) (Figure 1) resulted in several types of fragment ions. Two concurrent fragmentation patterns were detected. Detachment of carboxyl group from [LH_3_]^+^ caused the formation of an ion with *m/z* = 152.0. In the following step, an ion with *m/z* = 107.0 was obtained as a result of loss of groups: ethyl and hydroxyl. Further loss of the remaining substituent without the ring cleavage gave a protonated ion with *m/z* = 79.0. The competitive fragmentation of [LH_3_]^+^ was associated with the detachment of an [NH_3_CH(CH_3_)COOH]^+^ ion (*m/z* = 90.0), showing the highest relative intensity and formation of the ion with *m/z* = 108.0.

The ligand spectra were run at three pH values (2.0, 5.7, 9.6). The positive-ion spectra showed the presence of the [LH_3_]^+^ (*m/z* = 196.0) and its adduct [LH_3_ + NO_2_]^+^ (*m/z =* 242.0) in the acidic medium (Figure S3a,b). Deprotonated [LH_2_] as adduct [LH_2_ + Na + NO_2_]^+^ (*m/z =* 264.0) was also observed, showing the highest relative intensity at pH 9.6. Moreover, a number of signals have been detected for the fragment ions (see Figure S2) as well as their adducts (*m/z =* 111.0; 174.0; 211.0) (Figure S3).

In negative ion mode (Figure S4a,c), a trace presence of the deprotonated molecule [LH]^−^ (*m/z =* 194.0) was revealed. Alkalization led to an increase of the relative intensity of the ion and the loss of another hydrogen atom (*m/z =* 193.0), as confirmed by potentiometric studies (Figure S1). In addition, the adduct [LH + NO_2_]^−^ (*m/z =* 240.0) was registered at pH 2.0 and 5.7.

In an attempt to confirm the presence of the complexes in the Co(II)–AlaSal system, ESI-MS spectra at pH 6.3, 7.3, 9.0 and 10.3 were collected in positive and negative ion mode. The cobalt(II) aqua-ion ([Co(II) + 3NO_3_]^−^, *m*/*z =* 245) was the dominant form in the system at pH 6.3 (Figure S5a), which confirmed that metal–ligand complexing starts under these conditions. The complex with protonated ligand molecule [CoLH]^+^ (*m*/*z =* 253.0) and its adduct [CoLH + HNO_3_]^+^ (*m*/*z =* 316.0) were detected only at pH 6.3 (Figure S6a), just as in the potentiometric part. In turn, complex with *m/z =* 218.0, containing fragment ion *m/z* = 79.0 was observed across the whole tested pH range in positive ion mode (Figure S6). In addition, the bi-ligand complexes with protonated and also deprotonated ligand molecules were determined in both ion modes: [CoL_2_H]^−^ (*m*/*z =* 446.0), [CoL_2_ + Na]^−^ (*m*/*z =* 468.0), [CoL_2_H + NaOH]^−^ (*m*/*z =* 486.0), [CoL_2_H + Na + NO_3_]^−^ (*m*/*z =* 531.0), [CoL_2_H + 2HNO_3_]^−^ (*m*/*z =* 572.0) (Figure S5) and [CoL_2_H + 2H]^+^ (*m*/*z =* 448.0), [CoL_2_H + H + Na]^+^ (*m*/*z =* 470.0), [CoL_2_H + 2Na]^+^ (*m*/*z =* 492.0), [CoL_2_H + H + Na + HNO_3_]^+^ (*m*/*z =* 533.0) (Figure S6). Complexes were also observed with three AlaSal molecules, confirmed by the species distribution curves (Figure 3). Beside [CoL_3_H + 2Na + 2H + H_2_O]^+^ (*m*/*z =* 703.0) (Figure S6), other positive (*m*/*z =* 576.0, 618, 662; Figure S6) and negative (*m*/*z =* 615.0; Figure S5) adducts with fragment ions were detected.

### 2.3. UV/Vis Spectra

Three bands were observed at 238, 275, 291 nm for the absorption spectra of AlaSal within the 200–320 nm range (Figure S7a). No absorption above 320 nm was observed. The values of the molar absorption coefficients for the three [LH_2_], [LH]^–^ and [L]^2−^ ligand forms (Figure S7b) were calculated by HypSpec deconvolution at the studied pH range (1.90–11.30). Table 1 shows the maximum molar absorption coefficients for the particular forms along with the corresponding wavelengths. The isosbestic points were found at 221, 258, 280 nm and 263, 272 nm, which confirms the existence of equilibria between [LH_2_] and [LH]^−^ as well as [LH]^−^ and [L]^2−^, respectively.

The electronic absorption spectra in the presence of the metal ion indicate a blue shift of the *d–d* bands (to about 485 nm) relative to the Co(H_2_O)_6_^2+^ aqua-ion, 515 nm (*ε* = 4.6) [47] (Figure S8a). Above pH 5, the formation of two octahedral complexes [CoL] and [CoL_2_]^2−^ has been confirmed by HypSpec deconvolution (Figure S8b). These structures exist as the dominant forms in potentiometric titration (Figure 3). No other complexes have been identified, probably due to their lower concentration and simultaneous occurrence in a very similar pH range as [CoL] and [CoL_2_]^2−^ species. The maximum molar absorption coefficients for the complexes are given in Table 1, showing values of the same order as cobalt(II) complexes of the imines of amino acids derived from salicylaldehyde [48]. The charge transfer (CT) bands, observed within 300–400 nm, may be attributed to charge delocalization in the conjugated Schiff base ligand and assigned to a ligand-to-metal transition [42,49]. A weak shoulder is visible in this wavelength range, whose intensity increases above pH 6.65. This suggests deprotonation of the phenolic group and formation of [CoL], followed by [CoL_2_]^2−^ with the dianionic form of the ligand [50].

### 2.4. Kinetic Analysis

**Figure 4:**
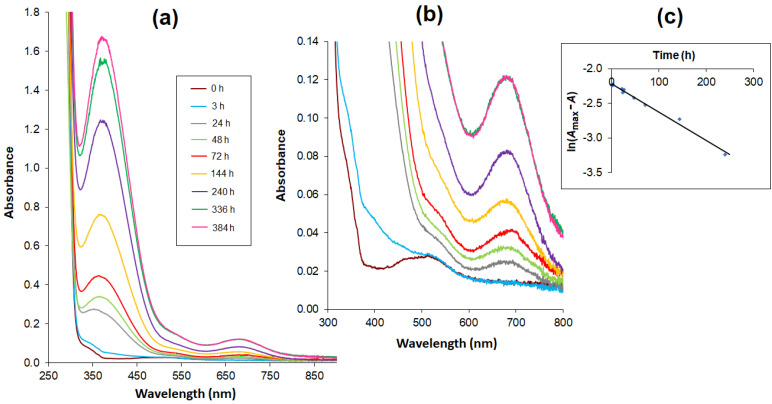
Absorption spectra of complexes in the Co(II) – AlaSal system (*C*_AlaSal_ = 2 × 10^−3^ mol L^−^^1^, at ligand-to-metal molar ratio 2:1) in 5 mM Tris-HCl/NaCl buffer at pH 7.2, recorded at consecutive time intervals within the wavelength range (**a**) 250–900 nm (**b**) 300–800 nm – extended part of spectra. (**c**) Time relationship of logarithmic values: ln(*A*_max_−*A*) at 683 nm.

The relationship between absorbance change and time was tested for the Co(II)–AlaSal system at a physiological pH (Figure 4a). Potentiometric titration found the main structures at a pH of about 7 to be the complexes [CoL], [CoL_2_H]^−^ as well as a small amount of [Co(H_2_O)_6_]^2+^ (Figure 3). This was also confirmed in the initial stage of the kinetic analysis. A characteristic band at the maximum absorbance of about 510 nm showed the presence of octahedral cobalt(II) complexes. After 24 h, a red shift of the peak was observed (Figure 4b), and the newly detected band at 683 nm could be attributed to four coordinate tetrahedral Co(II) complexes [27,47,51]. Therefore, it can be assumed that the structures of the [CoL] and [CoL_2_H]^−^ complexes changed from octahedral to tetrahedral. At the same time, a band at 370 nm appeared that could probably be attributed to a CT transition in tetrahedral complexes.

The time dependence of the absorbance increasing at 683 nm over 336 h (14 days) of measurements was essentially rectilinear. The obtained results were presented as a logarithmic function: ln(*A*_max_^−^*A*) = *f*(*t*), where the *A*_max_ value is sufficiently estimated on the basis of the absorbance value after 336 h (Figure 4c). The function shows a linear relationship with a determination coefficient of 0.9902, thus confirming a first-order reaction.

### 2.5. Biological Activity

Co(II) ions, AlaSal alone, and the complexes in the Co(II)–AlaSal system presented antibacterial and antifungal activity. For all formulations, minimal inhibitory concentration (MIC) and minimal bactericidal concentration (MBC) are shown in Table 2 along with MIC/MBC for standard antibiotics (gentamicin, amphotericin B and amoxicilicin). To facilitate the comparison of antimicrobial activity of the ligand, cobalt(II) and complexes with different molecular weights, MIC/MBC values were expressed in mM. Biological activity was studied for two Co(II)–AlaSal complex structures: octahedral (solution prepared directly) and tetrahedral (solution stored for two weeks). There were no differences in the MIC and MBC values for both evaluated geometric forms.

**Figure 5:**
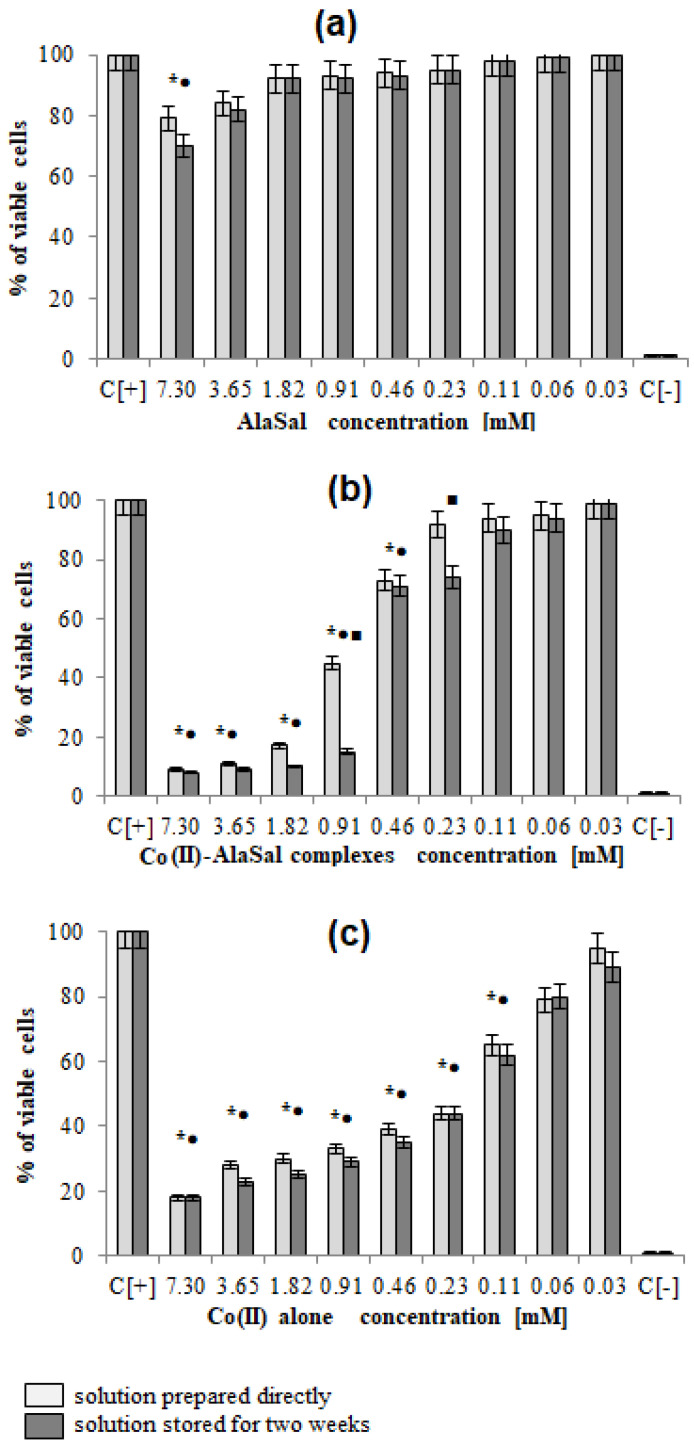
Cytotoxic effect of investigated compounds: (**a**) AlaSal, (**b**) Co(II)–AlaSal complexes (**c**) Co(II) alone towards L929 cells. The cytotoxicity was assessed by MTT [3-(4,5-dimethylthiazol-2-yl)-2,5-diphenyltetrazolium bromide)] reduction assay. The cell viability was calculated for four experiments including three repeats for each compound. Complete RMPI-1640 medium (cRPMI) was used as a positive control (C+) of cell viability (100% viable cells) and 0.03% H_2_O_2_ as a negative control (C^−^) of cell viability (100% dead inactive cells). Statistical significance: *•■ *p* < 0.05; * untreated cells vs. cells treated with tested solution (solution prepared directly); • untreated cells vs. cells treated with tested solution (solution stored for two weeks) ■ solution prepared directly vs. solution stored for two weeks.

**Figure 6:**
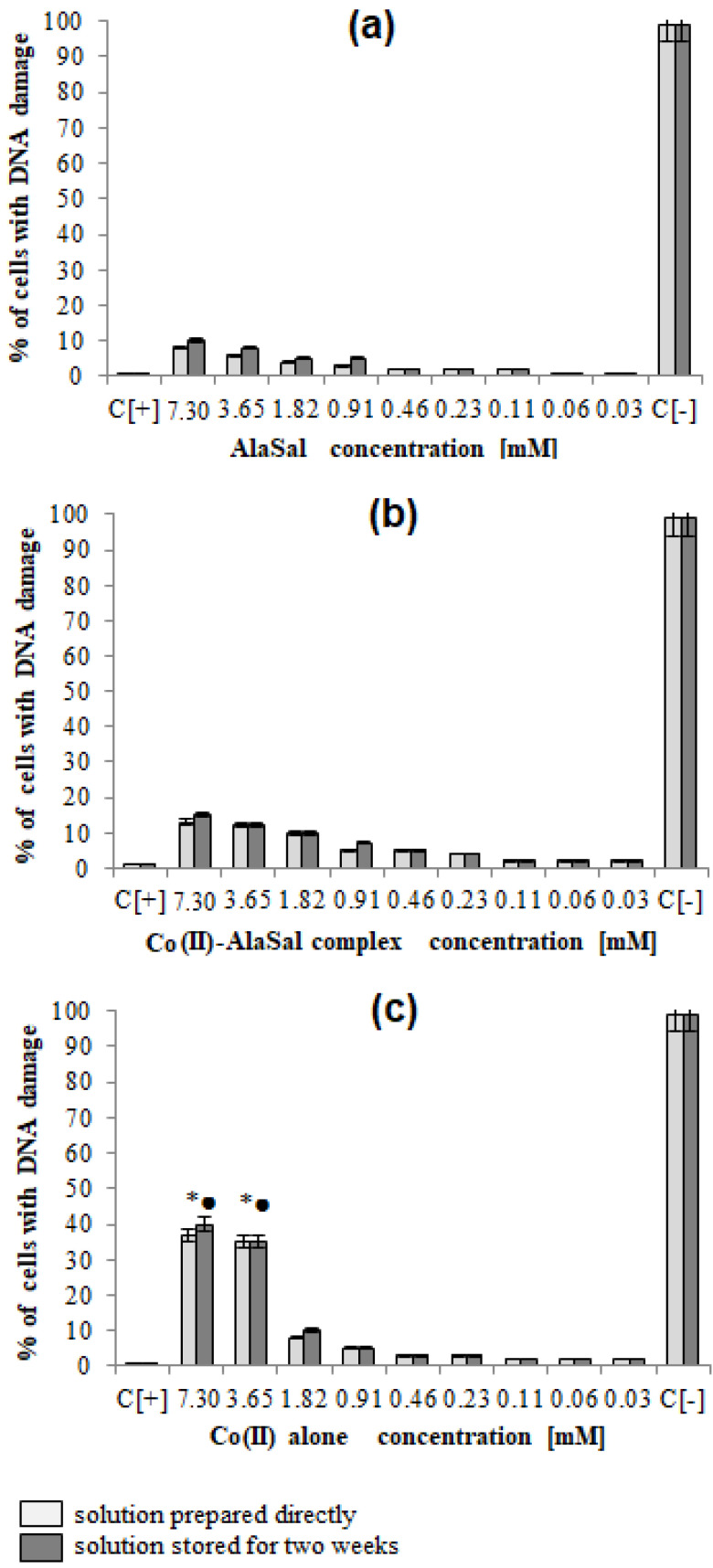
The percentage of L929 cells with damaged cell nuclei. The cells were stimulated for 24 h with: (**a**) AlaSal, (**b**) Co(II)–AlaSal complexes or (**c**) Co(II) alone and then stained by 4′,6-diamidino-2-phenylindole (DAPI). Statistical significance: *• *p* < 0.05; *untreated cells vs. cells treated with tested solution (solution prepared directly); • untreated cells vs. cells treated with tested solution (solution stored for two weeks).

**Figure 7:**
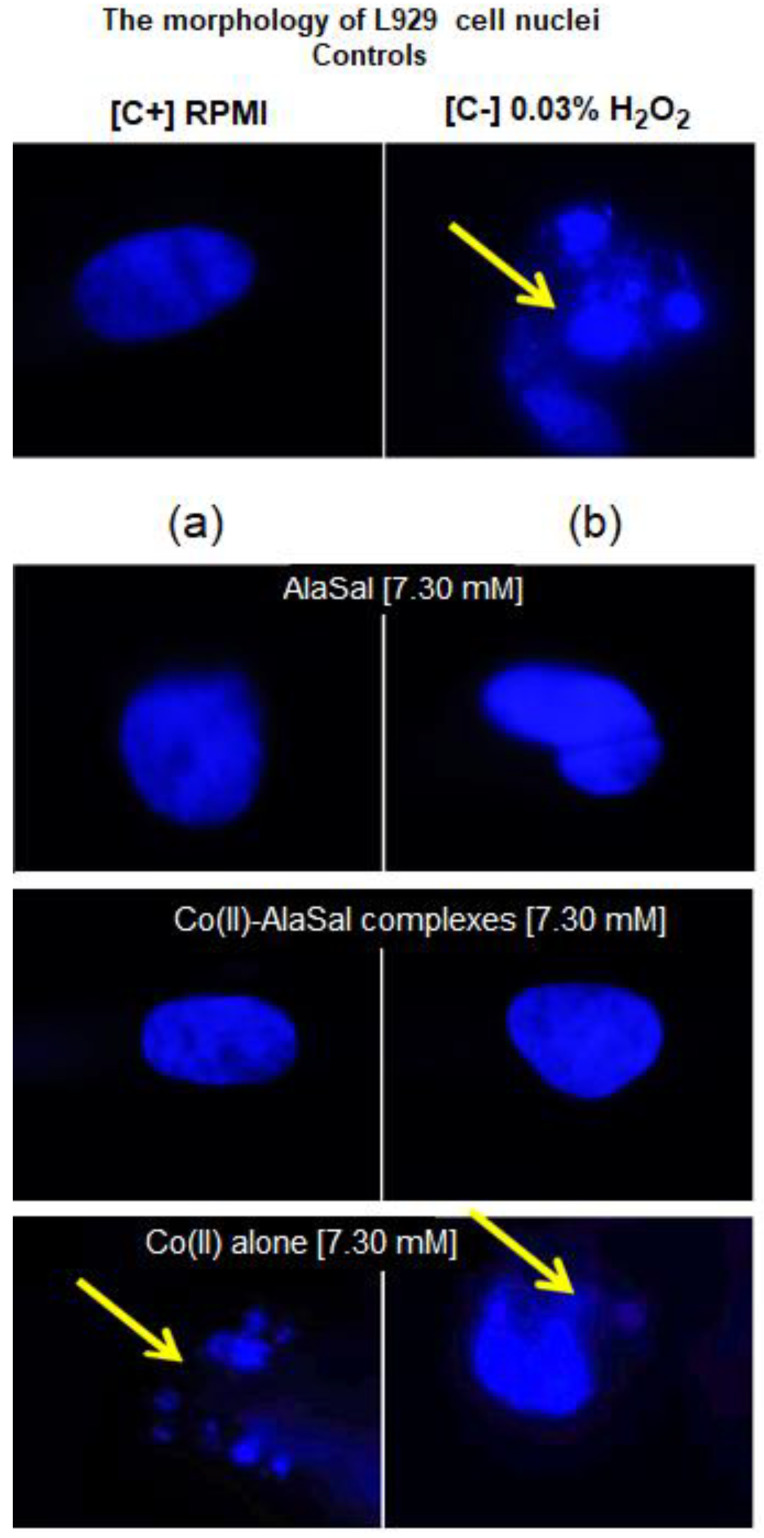
Microscopic images of L929 cells with a sign of cell nuclei damage. Cell cultures in complete RMPI-1640 medium (cRPMI) were used as positive control (C+): cells with no sign of cell nuclei damage; cells treated with 0.03% H_2_O_2_ were used as negative control (C−): cells with DNA damage. L929 cells stimulated with selected compounds: (**a**) solution prepared freshly or (**b**) solution stored for two weeks. The morphology of cell nuclei was assessed by 4′,6-diamidino-2-phenylindole (DAPI) staining. Samples were viewed under a fluorescent microscope (Axio Scope A1, Zeiss).

AlaSal exhibits the highest bacteriostatic and bactericidal activity against *P. aeruginosa* rods, for which MIC and MBC were 1.82 mM, while the MIC and MCB values 7.30 mM were found for *E. coli, S. aureus, S. epidermidis, H. pylori*, *E. faecalis* (Table 2). Most likely, the electron-donating amine nitrogen and hydroxyl oxygen at the *ortho* position of the benzene ring contribute to the antimicrobial activity [12,52,53,54,55]. The activities of AlaSal are found in the same order as observed in the case of the Schiff base ligands derived from L-glutamine and L-asparagine [56]. In turn, other reduced Schiff base amino acid ligands showed no inhibition against the selected microorganisms or were only effective against the Gram-negative bacteria [37]. For fungal strains, MIC equal to 1.82 mM was twice lower than MBC; this may be due to the different structures of the outer layers of bacterial and yeast cells, containing peptidoglycan or glucan, chitin or mannoproteins, respectively. AlaSal slightly diminished the growth of L929 fibroblasts within the concentration range 0.23–7.29 mM (5–25% of dead cells) (Figure 5a). Similarly, no significant anti-cellular toxicity was found for other Schiff bases derived from amino acids [10]. In the case of AlaSal stored for two weeks, the difference was statistically significant; however, neither the freshly prepared or stored AlaSal solutions induced cell nucleus blebbing, at any concentrations (Figure 6a, Figure 7).

Compared with the free ligand, complexation with a metal ion resulted in higher biological activity against the tested strains (except for *P. aeruginosa* and *E. coli*). As previously observed [29,42,57], the formation of the chelates improves their lipophilic nature, which increases permeability through the bacterial cell membrane. The lowest bacteriostatic concentration of Co(II)–AlaSal complexes, 1.82 mM (Table 2), was shown for *P. aeruginosa*, *E. faecalis, S. auresus, S. epidermidis* and *Candida* strains, while for *H. pylori* it was 3.65 mM and for *E. coli* 7.30 mM. MBC was the lowest for *P. aeruginosa* (1.82 mM), whereas for the remaining species of bacteria and fungi, excluding *E. coli*, the MBC was 3.65 mM. For *E. coli* it was above 7.30 mM. The complexes in the Co(II)–AlaSal system significantly diminished the ability of L929 cells to reduce MTT (5–92% of dead cells) within the range 0.23–7.29 mM (Figure 5b). The influence of the metal ion on the normal cell process may be the reason of the higher cytotoxic effect of the complexes then of the ligand alone [42]. The solution containing Co(II)–AlaSal complexes, stored for two weeks, had a stronger inhibitory effect at concentrations 0.23 and 0.91 mM than the freshly prepared material (Figure 5b). It is possible that the coordination geometry in the tetrahedral complex allows for greater exposure of the cobalt(II) center, thus permitting a higher binding capacity to new structures, compared to the octahedral complex, in which ligands can cause steric hindrance during interaction [10]. No signs of any cell nucleus vesicles were observed at any concentration of Co(II)–AlaSal complexes, regardless of how long the solutions were stored (Figure 6b, Figure 7). This means that the inhibition of cell growth was not correlated with irreversible damage of cell nuclei.

The most susceptible microbes to the bacteriostatic effect of Co(II) ions were *Candida* strains (0.23 mM), followed by *P. aeruginosa*, *S. aureus* and *S. epidemidis* (0.91 mM), while the least sensitive were *E. faecalis, E. coli* and *H. pylori* (1.82 mM). *E. coli* and Gram-positive bacteria demonstrated equal MBCs to MICs, while MBCs were a few or even several (for *Candida*) times higher than MICs for other bacterial strains. Co(II) ions strongly inhibited metabolic activity of mouse L929 fibroblasts within the range of 0.11–7.30 mM, which was assessed by the MTT reduction assay (Figure 5c) [29]. The Co(II) ions used at concentrations of 0.03 and 0.06 mM diminished the activity of treated versus untreated cells by about 5–20%. The storage time of the solutions did not affect the Co(II) ion activity towards L929 line (Figure 5c). Cells treated with Co(II) ions at the concentration range 3.65–7.30 mM were more likely to demonstrate morphological signs of cell apoptosis, compared to untreated cells (35–40% at *p* < 0.05) as indicated by blebbing nuclei (Figure 6b, Figure 7). However, the potentially adverse effects of Co(II) ions on eukaryotic cells exclude the possibility of its use in its bacteriostatic/bactericidal concentration. In vivo, at low pH of gastric juice, the uncomplexed cobalt(II) ion species will dominate (Figure 3a), which may be disadvantageous in the context of the medical use of the Co (II)–AlaSal system at such medium. However, taking into account the advantages of administering many medicinal products via the oral route, the use of new generation drug delivery systems may provide protection against low pH. Synthetic hydrogel-based delivery systems and particularly natural polymers such as alginate, hyaluronic acid and chitosan, are attractive matrices for oral drug delivery due to their biocompatibility, physiochemical properties, and mild gelation conditions. Recently, acrylic-based polymers have been extensively studied for the preparation of nanoparticles for oral drug administration [58]. In addition, transdermal use of the complex system can be another mode of its administration.

Significant quantitative differences in the MIC/MBC values can be seen between the tested formulations and standard antibiotics (Table 2). The high MIC/MBC values obtained for the Co(II)–AlaSal complexes are probably due to the fact that they were determined for two structures: [CoL] and [CoL_2_H]^−^ as well as a small percentage of cobalt(II) ions alone, existing in aqueous solution at pH 7.2 (Figure 3a). In addition, the ligand [LH_2_], with lower antimicrobial activity than complexes (Table 2), was the dominant form in the solution (Figure 3b). Other structures appeared in trace amounts, below 2.5% formation relative to metal ion or ligand. Nevertheless, the antibacterial and antimycotic activities of the Co(II)–AlaSal complexes on pathogens, which are the major etiologic agents of nosocomial infections, may be important in the fight against infections caused by multidrug resistant strains. It is also desirable to avoid damage of the nucleus in eukaryotic cells, which is ensured by the use of complexes within the tested concentration range (Figure 7).

## 3. Materials and Methods

### 3.1. Materials

*N*-(2-hydroxybenzyl)alanine (AlaSal) was synthesized as described previously [39]. Cobalt(II) nitrate hexahydrate and cobalt(II) perchlorate hexahydrate (Sigma-Aldrich), titrated with disodium salt of EDTA in the presence of murexide, were used as standard solutions. The alkali solutions (carbonate-free 0.1 M and 1.0 M NaOH), methanol and water (HPLC-grade) were purchased from J.T. Baker. The HNO_3_ and HClO_4_ solutions (Sigma-Aldrich) were standardized alkalimetrically and then determined by the Gran method [59]. Standard solutions of potassium nitrate(V) (J.T. Baker) and sodium perchlorate monohydrate (Sigma-Aldrich) were used to adjust the ionic medium. Tris-HCl (Sigma-Aldrich), sodium chloride (Chempur) and argon of high purity (Linde) were used.

The following materials were used in the biological studies: Mueller-Hinton liquid medium (Biocorp), *Brucella* medium (Biocorp), supplemented with 10% fetal bovine serum (FBS) (Sigma-Aldrich), RPMI-1640 with or without phenol red, amphotericin B, gentamicin, amoxicillin, penicillin, streptomycin, trypsin, MTT [(3-(4,5-dimethylthiazol-2-yl)-2,5-diphenyltetrazolium bromide)], 4′,6-diamidino-2-phenylindole (DAPI) (all from Sigma-Aldrich).

### 3.2. pH-Metric Titrations

The potentiometric titrations were performed using a Titrando 905 automatic titrator system (Metrohm) with a combined glass electrode (Metrohm LL Biotrode) filled with 3 M KCl electrolyte. The experiments were accomplished in aqueous solution at 25.0 ± 0.1 °C in a thermstatted closed vessel and ionic strength *I* = 0.1 M with KNO_3_. To ensure the absence of oxygen and carbon dioxide, pure argon was passed over the solution surface. The electrode was calibrated daily with NaOH on the hydrogen ion concentration using HNO_3_ solution [60]. All titrations were carried out in aqueous solutions in 4 mL samples.

The protonation constants of the AlaSal were determined by pH-metric titrations of various concentrations within 1.0–2.0 × 10^−2^ M, carried out in the pH range 2.0–11.5. The cobalt(II)–AlaSal system titrations were performed on solutions of metal concentrations of 4.0–10.0 × 10^−3^ M and ligand-to-metal molar ratios 2:1; 3:1; 5:1. The system was tested in a pH range of approximately 4.5–10.5. The formation constants of the aqua–hydroxo complexes of cobalt(II) were determined under the same conditions.

The concentration formation constants were calculated by the Hyperquad 2013 fitting procedure according to the formula: *β_mlh_* = [M*_m_*L*_l_*H*_h_*]/[M]*^m^*[L]*^l^*[H]*^h^* [61,62]. The ionic product of water p*K*_w_ = 13.77 [63] was included in the equilibrium model. The species distribution curves as a function of pH were calculated using the HySS 2009 [64].

### 3.3. Electrospray-Ionization Mass Spectrometry (ESI-MS) Measurements

Mass spectrometric data were obtained by means of a Varian 500-MS LC hexapole ion-trap mass spectrometer (Palo Alto, CA, USA). The experiments were performed in positive and negative ion-mode for AlaSal as well as the Co(II)–AlaSal system in 50/50% (*v*/*v*) methanol/water mixture, providing a more stable spray than water alone [65]. A background electrolyte was not added. The concentration of the ligand alone in the solution was 10^−2^ M, while the AlaSal concentration in the Co(II)–ligand system was 2 × 10^−2^ M at the molar ligand–metal ratio equal to 2:1. The samples were brought to various pH values known to achieve maximum concentrations of particular complexes and ionic forms of the ligand; these values were based on potentiometric species distribution graphs. All samples were introduced into the ESI-MS source by continuous infusion using an instrument syringe pump at a rate of 10 μL min^−1^. The ESI-source was operated at 5.00 kV and the capillary heater was set to 350 °C. The cone voltage was within the range 40–120 V.

### 3.4. Spectrophotometric Measurements

Electronic spectra under argon were recorded on a Cary 50 Bio spectrophotometer, slit width 1.5 nm, equipped with a fiber-optic device. This enabled the study of equilibrium systems spectrophotometrically, simultaneously with pH measurements controlled by a Molspin automatic titration kit with an InLab Semi-Micro combined polymer microelectrode (METTLER TOLEDO). Due to the highly disturbing absorption of the nitrate ion at about 300 nm, all the UV experiments were carried out in perchlorate medium, which was enabled by a combined polymer microelectrode. The pH and ionic strength (*I* = 1.0 M) were adjusted by HClO_4_ and NaClO_4_, respectively. The electrode was standardized with buffers at pH 4.00 and 7.00 before use. The fiber-optic probe, 5 mm long, corresponding to a path length of 1 cm, was dipped directly into the thermstatted titration vessel (constant temperature of 25.0 ± 0.1 °C kept constant). A stream of pure argon was passed over the sample surface to obtain the oxygen and carbon dioxide free solutions. After each addition of carbonate-free NaOH and an appropriate time delay to equilibrate the system, the pH and EMF (electromotive force) were measured. The spectrum was recorded with a slow scan (300 nm min^−1^) at selected pH values.

Perchlorate salts are potentially explosive and were handled only in small quantities with care.

At first, the AlaSal was tested at the wavelength range 200–400 nm in the absence of the metal (total concentration 2 × 10^−4^ M). For the Co(II) complexation equilibrium studies, a Co(II)–AlaSal solution was prepared in the 2:1 ligand–metal molar ratio, at total ligand concentration 2 × 10^−3^ M. At higher reagent concentrations, precipitation was observed during titration. The studies were carried out within the wavelength range 200–900 nm. The initial pH was set about 2.0 in all experiments. The molar absorbances of species have been calculated after deconvolution by HypSpec (part of Hyperquad 2008 suite, Protonic Software) [61].

### 3.5. Kinetics

The kinetics of complex structure changes in the Co(II)–AlaSal system was investigated by UV/Vis absorption spectroscopy (Cary 50 Bio spectrophotometer) using a fiber-optic device coupled with the Titrando 905 automatic titrator system and a InLab Semi-Micro combined polymer microelectrode (METTLER TOLEDO). The AlaSal solution (at a concentration of 2 × 10^−^^3^ M) in the presence of cobalt(II) ions at molar ligand–metal ratio of 2:1 was prepared in an aqueous 5 mM Tris-HCl/NaCl buffer. Then, the solution was placed in a closed thermstatted vessel at 25.0 ± 0.1 °C. Argon was constantly passed over the solution surface in the vessel to ensure the absence of oxygen and carbon dioxide, and the tested solution was adjusted to a pH of about 7.2 (with NaOH). Following this, UV/Vis spectra were recorded under anaerobic conditions at consecutive, long enough intervals, within 200–900 nm, simultaneously monitoring the pH values.

### 3.6. Biological Assays

In all the biological tests, three solutions were used: Co(II) ion solution, AlaSal solution and various complexes in the Co(II)–AlaSal system, freshly prepared and two weeks after preparation. The Co(II)–AlaSal system contained the molar ligand–metal ratio of 2:1. The solutions were made in the aqueous 5 mM Tris-HCl/NaCl buffer and adjusted to pH ~7.2 with NaOH. For testing, these ingredients were diluted to the following concentrations [mM]: 7.30, 3.65, 1.82, 0.91, 0.46, 0.23, 0.11, 0.06 and 0.03. The total concentration of the complexes in the Co(II)–AlaSal system was calculated using HySS simulation.

#### 3.6.1. Investigation of Antimicrobial Properties

The antimicrobial test was performed using reference bacterial strains from the American Type Culture Collection (ATCC), including Gram-positive strains: *Staphylococcus aureus* ATCC 6538 and ATCC 29213, *Staphylococcus epidermidis* ATCC 12228 and Gram-negative strains ATCC 29212, *Pseudomonas aeruginosa* ATCC 27853, *Helicobacter pylori* ATTC 700392, and three fungal strains: *Candida albicans* ATCC 10231, *Candida glabrata* ATCC 2001, and *Candida parapsilosis* ATCC 22019. One strain of *H. pylori* CCUG 17874 was from the Culture Collection, University of Gothenburg (CCUG), Sweden.

Antibacterial and antifungal activities were determined by the broth microdilution according to The European Committee on Antimicrobial Susceptibility (EUCAST) recommendations. Mueller-Hinton liquid medium (pH ~ 7.2) was used for bacteria with limited nutritional requirements, while *Brucella* medium enriched with 10% (*v*/*v*) fetal bovine serum (FBS) was used for bacteria with high nutritional requirements (*H. pylori* ATTC 700392 and CCUG 17874). Liquid medium without phenol red RPMI-1640 (pH ~ 7.2) was used for the fungal strains. Two-fold series dilutions of the studied formulations in the growth medium were prepared in the 96-well sterile microtiter plates (Becton-Dickinson GmbH, Heidelberg, Germany).

Inocula were freshly prepared and standardized as microbial suspensions (McFarland scale) containing 1 × 10^8^ colony forming units (CFU mL^−1^), added at a volume of 10 μL to the wells of the microtiter plate, together with the serial dilutions of the compounds in the growth medium. After 24 h of incubation at 37 °C the microbial growth was evaluated spectrophotometrically at 595 nm using a Victor2 spectrophotometer (Wallac, Turku, Finland). Microtiter plates with *H. pylori* were incubated under microaerophilic conditions at 37 °C for three days. The antimicrobial activity of the formulations, expressed in mM, was evaluated based on their minimal inhibitory concentrations (MIC) and minimal bactericidal concentrations (MBC). The lowest concentration resulting in total growth inhibition was taken as MIC. To determine MBC, 10 μL of the culture were collected from each well, where no visible growth of microorganisms was recorded and plated onto the surface of Columbia Agar, supplemented with 7% (*v*/*v*) sheep’s blood. The cultures were incubated for 24 h at 37 °C and no microbial growth indicated bactericidal activity of the tested compounds. Plates with *H. pylori* were incubated under microaerophilic conditions at 37 °C for three days. The above-mentioned tests were performed in three independent experiments. Amphotericin B, gentamicin and amoxicillin were used as standard antimicrobials.

#### 3.6.2. Cell Cultures

The L929 mouse fibroblasts (LGC Standards, Middlesex, UK) were used for in vitro cytotoxicity testing. The cells were maintained under standard conditions (37 °C, 5% CO_2_) in 25-cm^2^ tissue culture flasks in RPMI 1640 medium supplemented with 10% fetal bovine serum (FBS) and antibiotics: 100 U mL^−1^ penicillin and 100 µg mL^−1^ streptomycin. To obtain the cell suspension for cytotoxicity assay or to start a new culture, confluent monolayers were treated with 0.25% trypsin solution, washed and subcultured at a cell density of 1 × 10^8^ cells mL^−1^. Cell cultures were supplemented with fresh medium two or three times per week to maintain their growth in the log phase. Prior to experiments, the viability of the cells was assessed by exclusion of trypan blue dye and remained within the range 93–95%.

##### Measurements of Cellular Metabolic. Activity and Global Growth Inhibition

For testing the influence of compounds used in this study on cellular metabolic activity, L929 cells were seeded into 96-well plates (2 × 10^5^ cells/well). Tested compounds were diluted serially in RPMI-1640 medium and added to the cells (100 μL/well), then incubated under standard conditions for 24 h. The potential harmful effect of studied samples on L929 cells was assessed by screening the ability of the cells to reduce MTT [(3-(4,5-dimethylthiazol-2-yl)-2,5-diphenyltetrazolium bromide)] as recommended by the Food and Drug Administration and the International Organization for Standardization. Fresh MTT solution (5 mg mL^−1^ in sterile PBS) was added to each well and the plates were incubated for 4 h at 37 °C, 5% CO_2_. Formazan crystals were dissolved with acidic isopropanol (0.1 M HCl in absolute isopropanol). Optical densities (OD) were then measured at the reference 570 nm wavelength using a Victor2 spectrophotometer (Wallac, Turku, Finland). Results are presented as the mean percentage ± standard deviation (SD) of the treated cells versus untreated cells (used in triplicates), in four independent experiments. The effectiveness of MTT reduction was calculated based on the following formula: MTT reduction by treated cells versus untreated cells (%) = (absorbance of treated cells/absorbance of untreated cells × 100%) − 100%.

##### DAPI Staining of Cell Nuclei

The nuclei of L929 cells were stained as previously described [66] using a fluorescent dye 4’,6-diamidino-2-phenylindole (DAPI), which has a strong affinity to the AT base pair in DNA. Cells, after 24 h of stimulation with selected compounds: Co(II) ions, AlaSal and the Co(II)–AlaSal complexes in decreasing concentrations, were fixed with 4% formaldehyde, and stained with DAPI solution (2.5 µg mL^−1^) for 15 min, at room temperature and the viewed under a fluorescent microscope (Zeiss, Axio Scope, A1), at a wavelength of 358 nm (excitation) and 461 nm (emission). A percentage of cells with blebbing nucleus was assessed.

#### 3.6.3. Statistical Analysis

The data were compared using the Kruskal–Wallis test. Statistical significance was accepted at a *p*-value < 0.05. Data are presented as mean values ± SD. For statistical analysis the STATISTICA 12 PL software was used (Stat Soft, Poland).

## 4. Conclusions

Potentiometric measurements allowed the molecular formulas of six complexes formed in the Co(II)–AlaSal aqueous system to be determined, and for the overall stability constants of all the species to be to calculated. The determined p*K*_a_ values of AlaSal confirmed the tridentate nature in {O^-^_phenolic_,N,O^-^_carboxyl_} chelation mode. The Co(II)–AlaSal complexes demonstrated higher stability constants than the analogous species with other metal ions, indicating coordination in the equatorial plane. Moreover, cobalt(II) complexes with a protonated phenolic group in the ligand molecule were formed in an aqueous solution. The obtained complexes were also confirmed by ESI-MS studies at various pH levels. The results of UV/Vis measurements indicate the formation of two octahedral structures: [CoL] and [CoL_2_]^2−^, predominating as the most abundant ones in potentiometric titrations. At pH of about 7, which is particularly important for biological studies, the kinetic analysis showed a change in the structures of the cobalt(II) complexes from octahedral to tetrahedral according to the first-order time dependence.

The [CoL] and [CoL_2_H]^−^ complexes present in the equilibrium mixture under physiological pH, as well as the AlaSal and Co(II) ion alone, revealed antimicrobial activities against the tested strains: *Enterococcus faecalis*, *Staphylococcus aureus*, *Staphylococcus epidermidis*, *Pseudomonas aeruginosa*, *Escherichia coli*, *Helicobacter pylori*, *Candida*. The presence of the ligand electron-donating groups is most likely one of the factors favoring these properties. The Co(II)–AlaSal complexes, due to their lipophilic nature and increased permeability through the bacterial cell membrane, exhibit better biological activity for most strains than the free ligand. In addition, tetrahedral structures showed stronger anti-cellular toxicity than octahedral complexes, most probably because of the higher exposure of the cobalt(II) center. Regardless of the concentrations of used solutions, AlaSal alone and cobalt(II) complexes did not induce irreversible damage of cell nuclei. Therefore, these compounds can be used as the basis for the development of new preparations used in the fight against infections caused by strains resistant to other drugs. Thus, it seems advisable in further experimental studies to provide evidence for the role of individual complexes of the equilibrium mixture at physiological pH in biological activity. The treatment of eukaryotic cells only by Co(II) ions causes the formation of nuclear vesicles, suggesting it is not suitable for use as an antimicrobial preparation.

## Figures and Tables

**Table 1:** Decimal logarithms of overall protonation and formation constants in the Co(II)−AlaSal system, β_mlh_ = [M_m_L_l_H_h_]/[M]^m^[L]^l^[H]^h^ at 25.0 ± 0.1 °C, I = 0.1 (KNO_3_) and UV/Vis spectral data. Standard deviations in parentheses after overall protonation and stability constants refer to random errors only.

Species	log_10_ *β_mlh_*	Stepwise Dissociation Constants	Related Constants	*λ*_max_ (*ε*_max_)
[L]^2−^				236 (8.2 × 10^3^)
				292 (3.5 × 10^3^)
[LH]^−^	10.73(1) (OH)	p*K*_a3_ 10.73		237 (6.1 × 10^3^)
				291 (2.6 × 10^3^)
[LH_2_]	19.36(1) (NH_2_^+^)	p*K*_a2_ 8.63 ^2^		~240^sh^ (4.1 × 10^3^)
				274 (2.1 × 10^3^)
[LH_3_]^+^	21.66(2) (COOH)	p*K*_a1_ 2.30 ^3^		~298^sh^ (1.6 × 10^3^)
*σ*; *n*^1^	5.82; 804			
[CoL]	7.98(1)			508 (21)
[CoL_2_]^2−^	13.35(2)			483 (31)
[CoL_3_]^4−^	16.39(4)			
[CoLH]^+^	13.64(10)		2.92 ^4^	
	21.78(2)		11.06 ^5^	
[CoL_2_H]^−^	26.35(4)		15.63 ^6^	
[CoL_3_H]^3−^				
*σ*; *n*^1^	5.85; 455			

^1^*σ*—the value of the normalized sum of squared residuals; *n*—number of titration points; ^2^ p*K*_a2_ = $\log_{10}K_{{\mathrm{LH}}_{2}}^{\mathrm{LH}}=\log\beta_{{\mathrm{LH}}_{2}}-\log\beta_{\mathrm{LH}}$. ^3^ p*K*_a1_ = $\log_{10}K_{{\mathrm{LH}}_{3}}^{{\mathrm{LH}}_{2}}=\log\beta_{{\mathrm{LH}}_{3}}-\log\beta_{{\mathrm{LH}}_{2}}$. ^4^ $\log_{10}K_{\mathrm{CoLH}}^{\mathrm{Co}}=\log\beta_{\mathrm{CoLH}}-\log\beta_{\mathrm{LH}}$. ^5^ $\log_{10}K_{{\mathrm{CoL}}_{2}H}^{\mathrm{Co}}=\log\beta_{{\mathrm{CoL}}_{2}H}-\log\beta_{\mathrm{LH}}$. ^6^ $\log_{10}K_{{\mathrm{CoL}}_{3}H}^{\mathrm{Co}}=\log\beta_{{\mathrm{CoL}}_{3}H}-\log\beta_{\mathrm{LH}}$.

**Table 2:** Antimicrobial activity of tested compounds, prepared directly and stored for two weeks, shown as minimal inhibitory concentration (MIC) and minimal bactericidal concentration (MBC). Gentamicin, amoxicillin and amphotericin B used as antibacterial and antifungal reference substances, respectively; (-) not tested.

Microorganism	MIC/MBC (mM)						MIC = MBC (mM)		
	AlaSal		Co(II) Alone		Co(II)–AlaSal Complexes		Gentamicin	Amphotericin B	Amoxicillin
	MIC	MBC	MIC	MBC	MIC	MBC			
Gram-negative bacteria									
*Pseudomonas aeruginosa* ATCC 27853	1.82	1.82	0.91	1.82	1.82	1.82	<0.008	-	-
*Escherichia coli* ATCC 25922	7.30	>7.30	1.82	1.82	7.30	>7.30	<0.004	-	-
*Helicobacter pylori* CCUC 17874	7.30	>7.30	1.82	>7.30	3.65	3.65	-	-	<0.001
*Helicobacter pylori* ATCC 700392	7.30	>7.30	1.82	>7.30	3.65	3.65	-	-	<0.001
Gram-positive bacteria									
*Enterococcus faecalis* ATCC 29212	7.30	>7.30	1.82	1.82	1.82	3.65	<0.26	-	-
*Staphylococcus aureus* ATCC 29213	7.30	>7.30	0.91	0.91	1.82	3.65	<0.002	-	-
*Staphylococcus aureus* ATCC 6538	7.30	>7.30	0.91	0.91	1.82	3.65	<0.002	-	-
*Staphylococcus epidermidis ATCC 12228*	7.30	>7.30	0.91	0.91	1.82	3.65	<0.002	-	-
Fungi									
*Candida albicans* ATTC 10231	3.65	>7.30	0.23	3.65	1.82	3.65	-	<0.001	-
*Candida glabrata* ATCC 2001	3.65	>7.30	0.23	3.65	1.82	3.65	-	<0.001	-
*Candida parapsilosis* ATCC 22019	3.65	>7.30	0.23	7.30	1.82	3.65	-	<0.001	-

## Supplementary Materials

The following are available online, Figure S1: Species distribution curves, Figures S2–S6: mass spectra, Figures S7 and S8: electronic absorption spectra.
