# Supplementary material for: Equilibria in Aqueous Cobalt(II)—Reduced Schiff Base N-(2-hydroxybenzyl)alanine System: Chemical Characterization, Kinetic Analysis, Antimicrobial and Cytotoxic Properties
Source: Molecules. 2020 Jul 30;25(15):3462. doi: 10.3390/molecules25153462 (PMC7436002; doi:10.3390/molecules25153462)
Supplement: Supplementary file 1 [file molecules-25-03462-s001.pdf]

Supplementary Materials for the paper

Equilibria in Aqueous Cobalt(II) – Reduced Schiff Base *N*-(2-hydroxybenzyl)alanine System: Chemical Characterization, Kinetic Analysis, Antimicrobial and Cytotoxic Properties

Magdalena Woźniczka [1,*], Manas Sutradhar [2], Armando J.L. Pombeiro [2], Mirosława Świątek [1], Marek Pająk [1], Joanna Gądek-Sobczyńska [1], Magdalena Chmiela [3], Weronika Gonciarz [3], Beata Pasternak [4] and Aleksander Kufelnicki [1]

Page 3. **Figure S1.** Species distribution curves as a function of pH for AlaSal; C_{AlaSal} = 2.0 × 10^{-2} M.

Page 4. **Figure S2.** Tandem mass spectrum of $[LH_3]^+$ – *m/z* = 196.0, C_{AlaSal} = 1.0 × 10^{-2} M.

Page 5. **Figure S3.** ESI-MS spectra for ligand in positive ion-mode at various pH, C_{AlaSal} = 1.0 × 10^{-2} M. Explanation of the adduct signal described in the text:
m/z = 111.0 [fragment ion *m/z* = 79 + $CH_3OH]^+$
m/z = 174.0 [deprotonated fragment ion *m/z* = 152 + $Na]^+$
m/z = 211.0 [fragment ion *m/z* = 152 + NaOH + $H_2O]^+$.

Page 6. **Figure S4.** ESI-MS spectra for ligand in negative ion-mode at various pH, C_{AlaSal} = 1.0 × 10^{-2} M.

Page 7. **Figure S5.** Negative-ion ESI-MS spectra for the complexes formed in the $Co(NO_3)_2$/AlaSal system at ligand-to-metal molar ratio 2:1, at various pH, C_{AlaSal} = 2 × 10^{-2} M. Explanation of the signal described in the text:
m/z = 615.0 [CoL_2H + deprotonated fragment ion *m/z* = 107 + $HNO_3]^-$.

Page 8. **Figure S6.** Positive-ion ESI-MS spectra for the complexes formed in the $Co(NO_3)_2$/AlaSal system at ligand-to-metal molar ratio 2:1, at various pH, C_{AlaSal} = 2 × 10^{-2} M. Explanation of the signal described in the text:
m/z = 218.0 [Co(II) + fragment ion *m/z* = 79 + 2OH + $NO_2]^+$
m/z = 576.0 [CoL_2H + fragment ion *m/z* = 107 + $Na]^+$
m/z = 618.0 [CoL_2H + fragment ion *m/z* = 108 + H + $HNO_3]^+$
m/z = 662.0 [CoL_2H + fragment ion *m/z* = 152 + H + $HNO_3]^+$.

Page 9. **Figure S7. (a)** UV spectra of AlaSal during titration within the pH range 1.90–11.30, $C_{AlaSal} = 2 \times 10^{-4}$ M; **(b)** Molar absorption coefficients (ε) of various ligand ionic forms.

Page 10. **Figure S8. (a)** UV/Vis spectra during titration of the Co(II) – AlaSal system at ligand-to-metal molar ratio 2:1, pH range 1.80–11.43; $C_{AlaSal} = 2 \times 10^{-3}$ M. **(b)** Molar absorption coefficients (ε) of the two complexes accepted by HypSpec deconvolution.

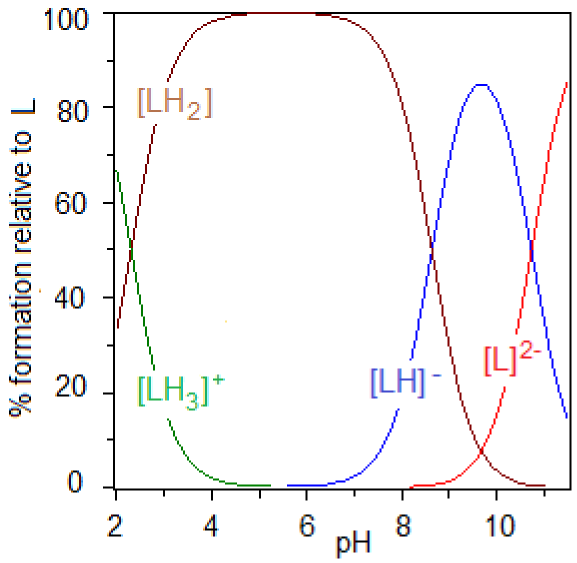

Figure S1. Species distribution curves as a function of pH for AlaSal; $C_{AlaSal} = 2.0 \times 10^{-2}$ M.

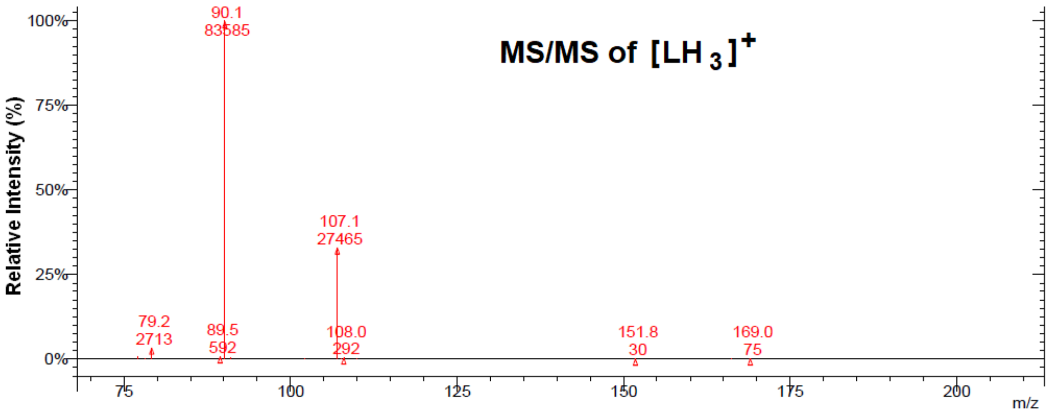

Figure S2. Tandem mass spectrum of $[LH_3]^+$ – m/z = 196.0, C_{AlaSal} = 1.0 × 10^{-2} M.

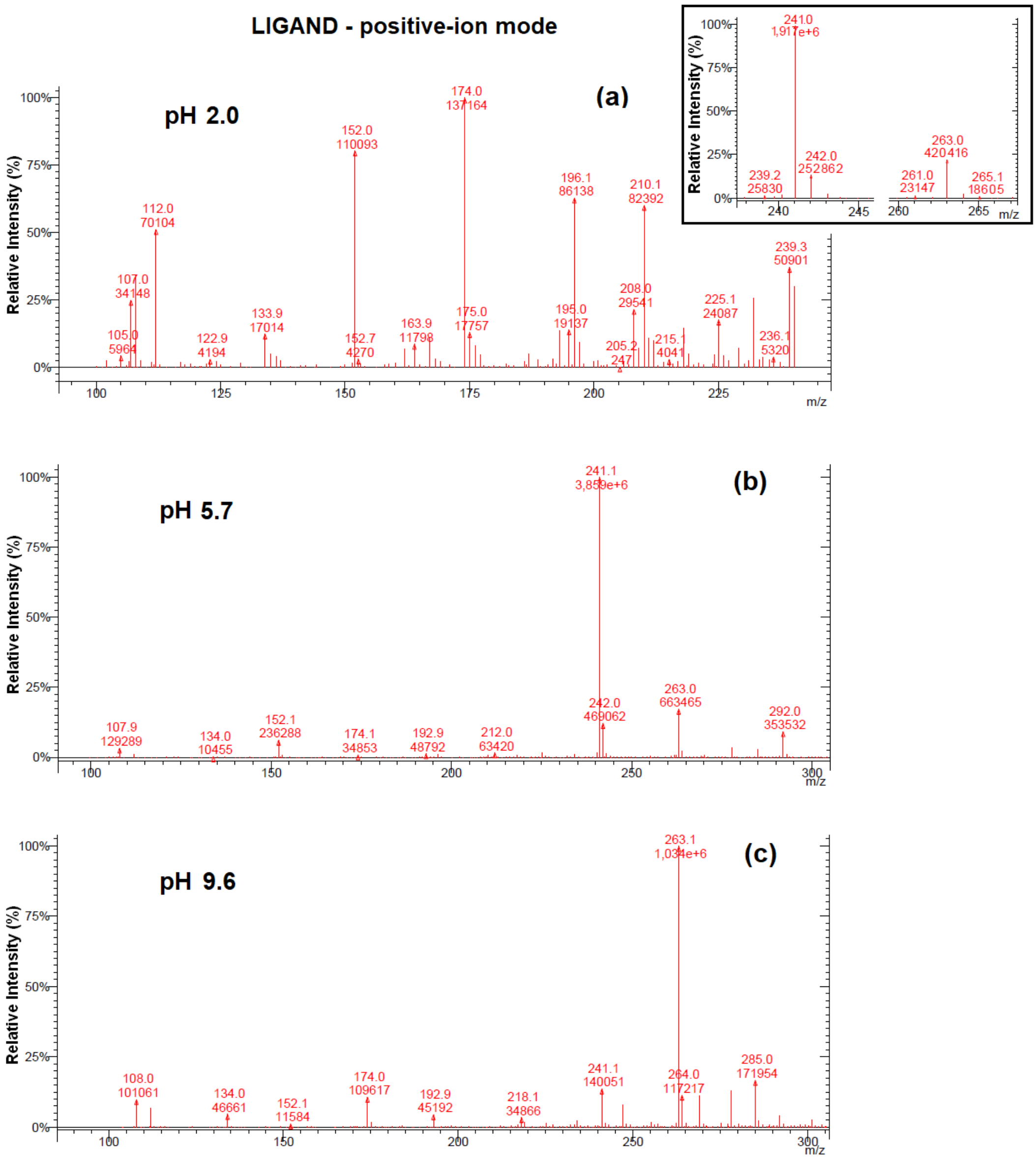

Figure S3. ESI-MS spectra for ligand in positive ion-mode at various pH, $C_{AlaSal} = 1.0 \times 10^{-2}$ M. Explanation of the adduct signal described in the text:
m/z = 111.0 [fragment ion *m/z* = 79 + CH_3OH]$^+$
m/z = 174.0 [deprotonated fragment ion *m/z* = 152 + Na]$^+$
m/z = 211.0 [fragment ion *m/z* = 152 + NaOH + H_2O]$^+$.

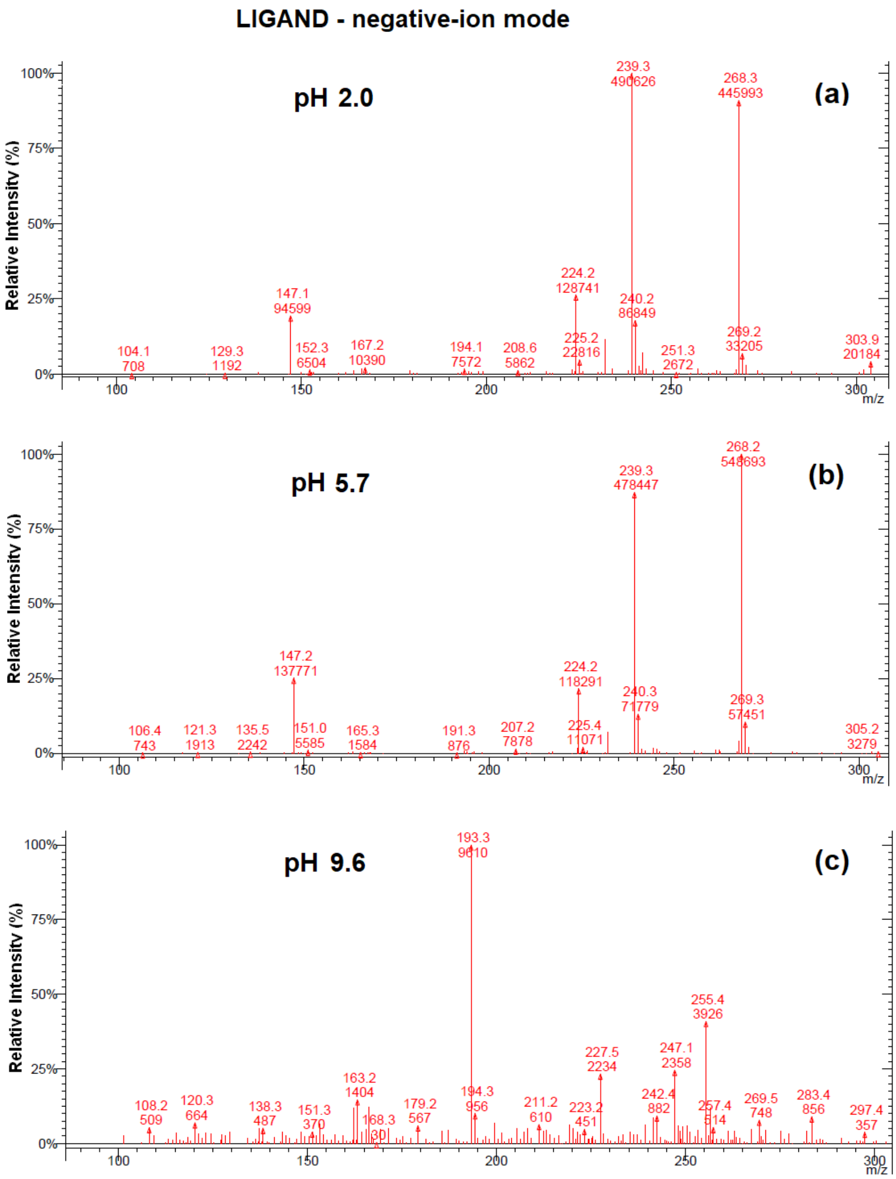

Figure S4. ESI-MS spectra for ligand in negative ion-mode at various pH, C_{AlaSal} = 1.0 × 10^{-2} M.

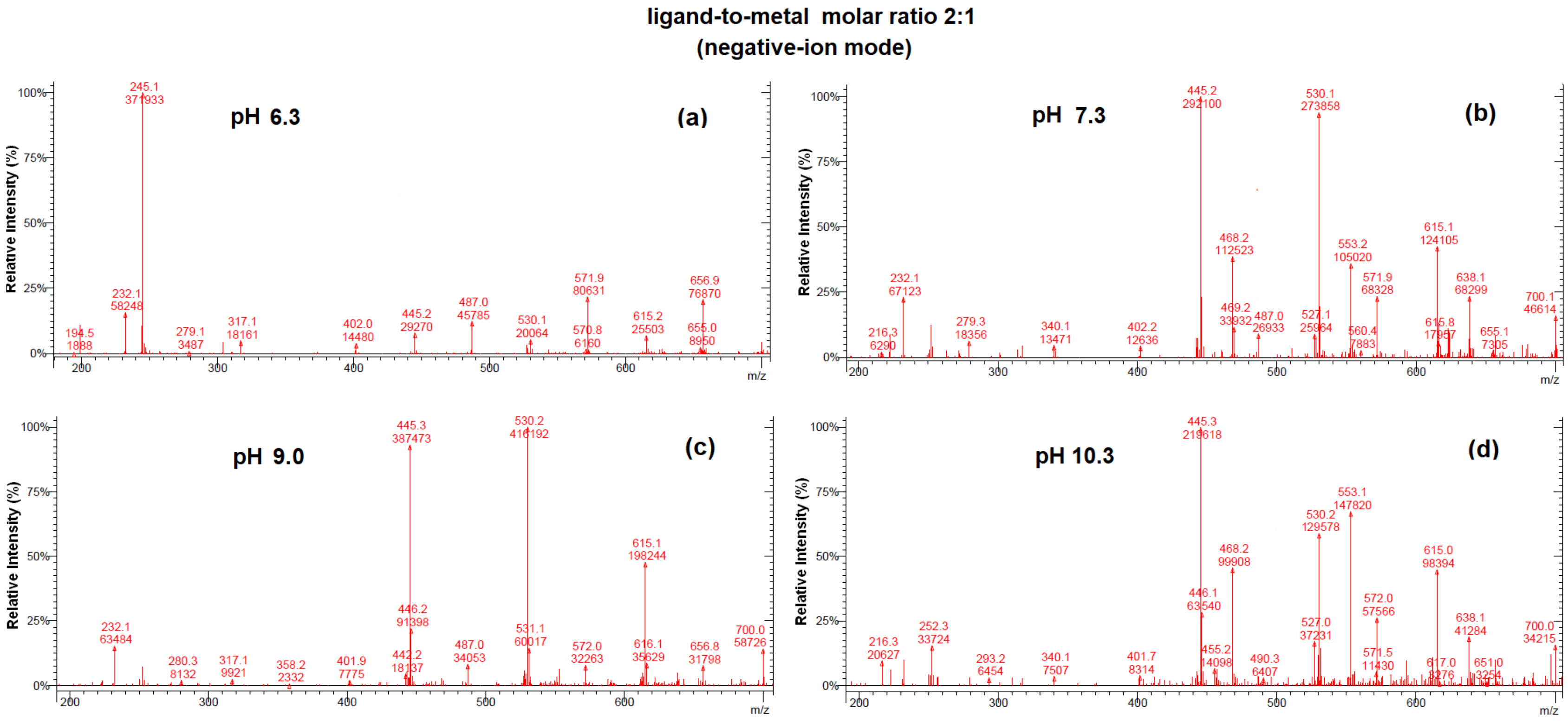

Figure S5. Negative-ion ESI-MS spectra for the complexes formed in the $Co(NO_3)_2$/AlaSal system at ligand-to-metal molar ratio 2:1, at various pH, $C_{AlaSal} = 2 \times 10^{-2}$ M. Explanation of the signal described in the text:
m/*z* = 615.0 [CoL_2H + deprotonated fragment ion *m*/*z* = 107 + HNO_3]$^-$.

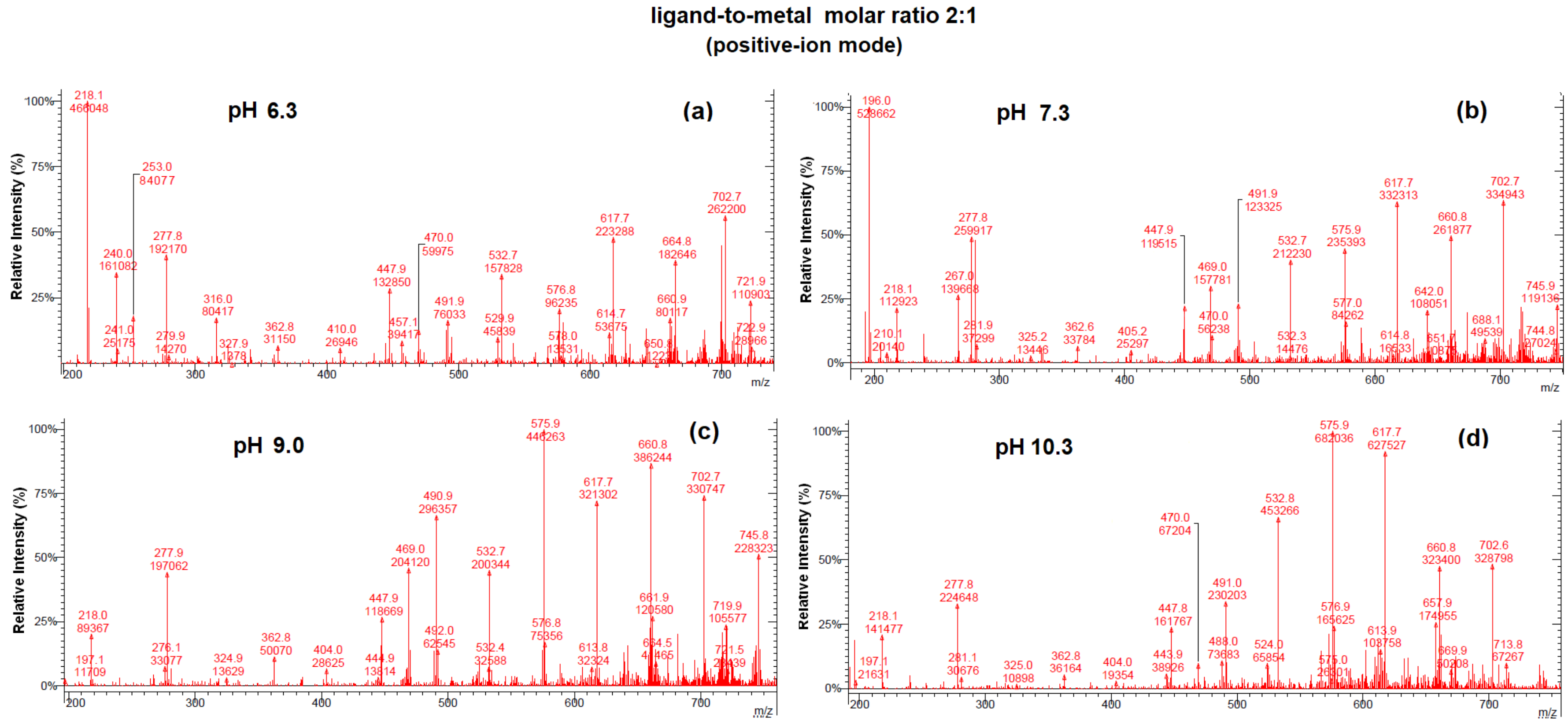

Figure S6. Positive-ion ESI-MS spectra for the complexes formed in the $Co(NO_3)_2$/AlaSal system at ligand-to-metal molar ratio 2:1, at various pH, $C_{AlaSal} = 2 \times 10^{-2}$ M. Explanation of the signal described in the text:

m/z = 218.0 [Co(II) + fragment ion m/z = 79 + 2OH + NO_2]$^+$

m/z = 576.0 [CoL_2H + fragment ion m/z = 107 + Na]$^+$

m/z = 618.0 [CoL_2H + fragment ion m/z = 108 + H + HNO_3]$^+$

m/z = 662.0 [CoL_2H + fragment ion m/z = 152 + H + HNO_3]$^+$.

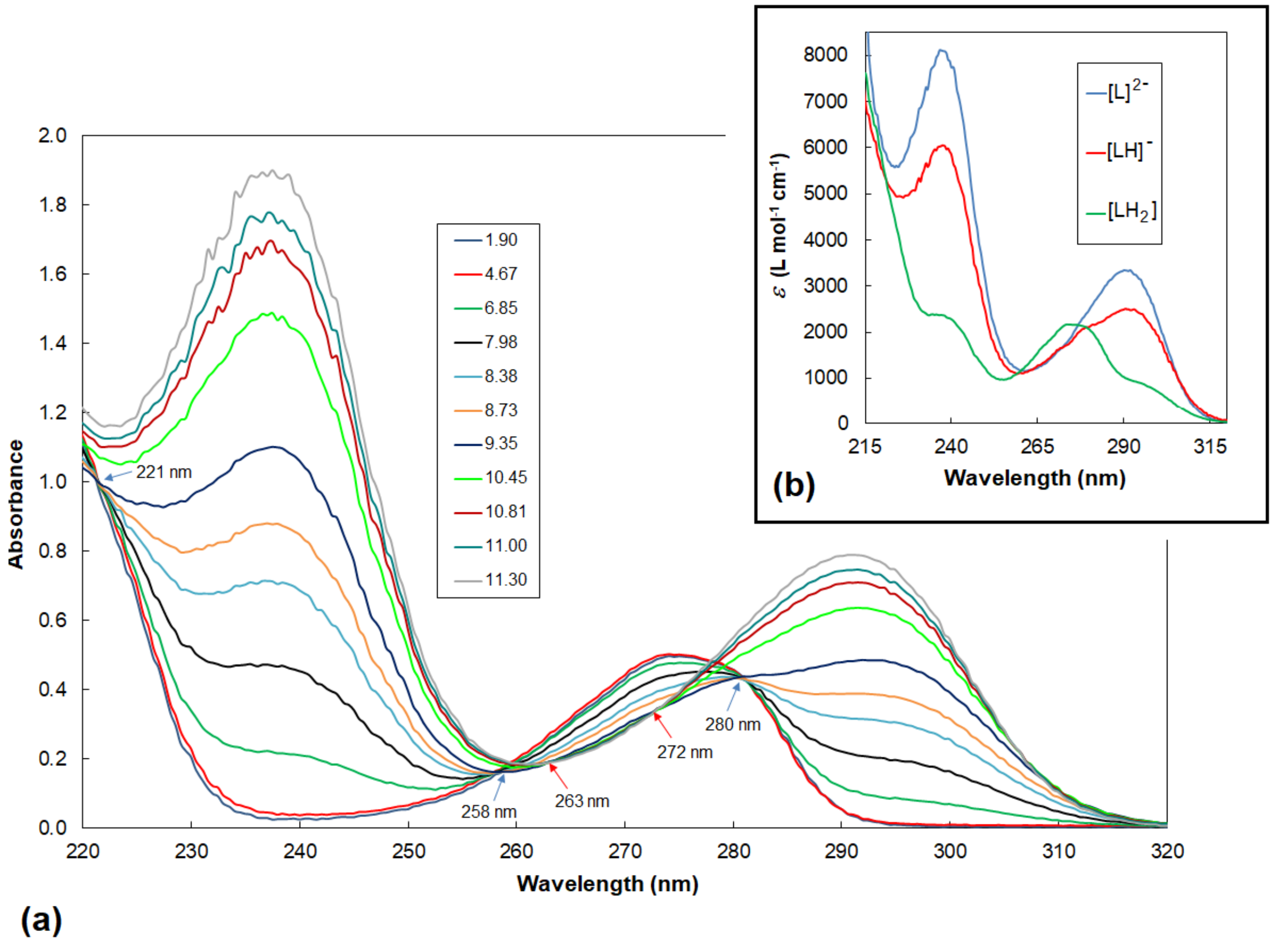

Figure S7. (a) UV spectra of AlaSal during titration within the pH range 1.90–11.30, C_{AlaSal} = 2×10^{-4} M; **(b)** Molar absorption coefficients (ε) of various ligand ionic forms.

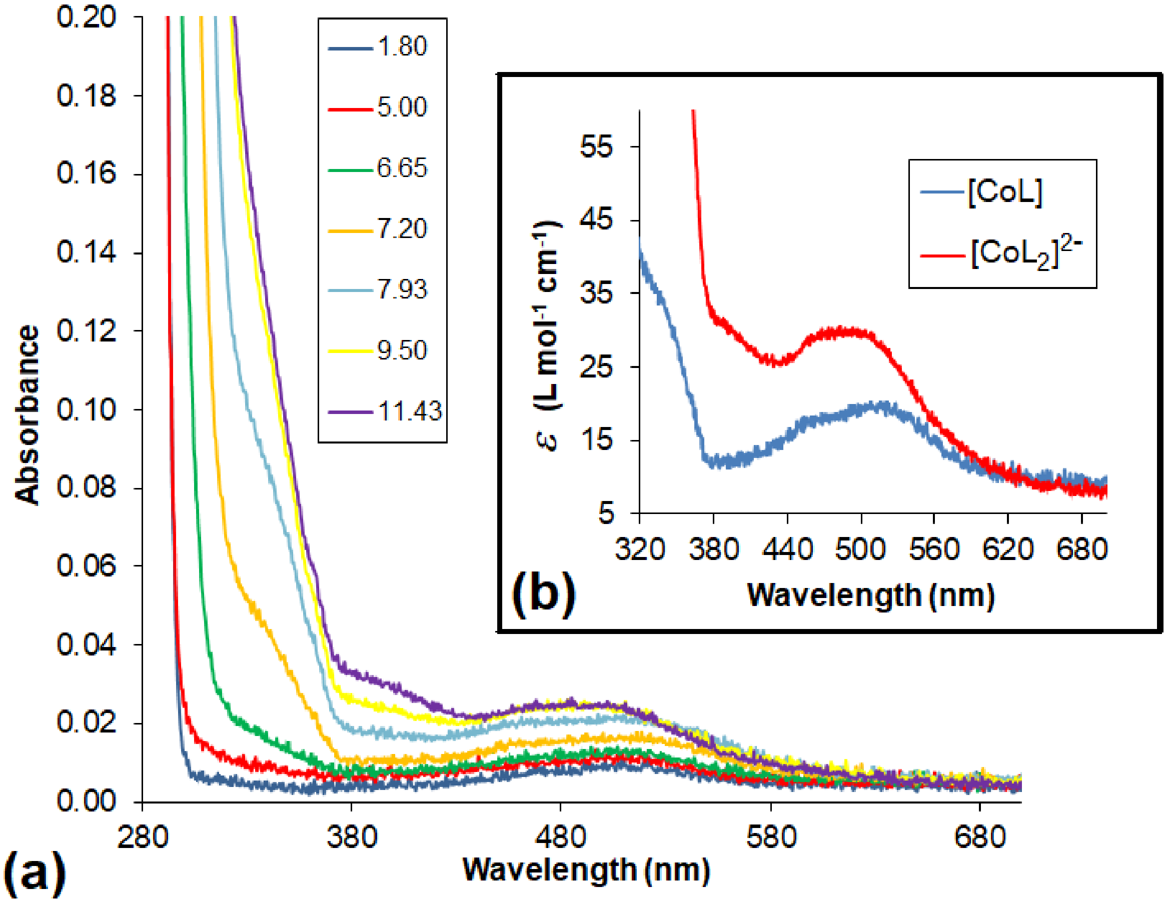

Figure S8. **(a)** UV/Vis spectra during titration of the Co(II) – AlaSal system at ligand-to-metal molar ratio 2:1, pH range 1.80–11.43; $C_{AlaSal} = 2 \times 10^{-3}$ M. **(b)** Molar absorption coefficients (ε) of the two complexes accepted by HypSpec deconvolution.
